# P-1477. Epidemiological and Economic Impact of Modifying the Meningococcal Vaccination Schedule for Adolescents

**DOI:** 10.1093/ofid/ofaf695.1663

**Published:** 2026-01-11

**Authors:** Thomas Shin, Olivier Cristeau risteau, Emilie Clay, Angie Upegui, Rachel Dawson, David P Greenberg, Maureen P Neary

**Affiliations:** Sanofi Pasteur, Toronto, ON, Canada; Clever-Access, Paris, Ile-de-France, France; Clever-Access, Paris, Ile-de-France, France; Sanofi, Lyon, Auvergne, France; Sanofi, Lyon, Auvergne, France; Sanofi Pasteur, Toronto, ON, Canada; Sanofi, Lyon, Auvergne, France

## Abstract

**Background:**

Invasive meningococcal disease (IMD) is a serious and life-threatening bacterial infection caused by *Neisseria meningitidis.* The quadrivalent meningococcal conjugate vaccine (MenACWY) program for adolescents and young adults (AYAs) in the US was first implemented in 2005 and has evolved as new vaccines and data have become available. Prior studies have demonstrated the value of the current vaccination scheme (CVS, 2 Q = MenACWY doses) vs. no vaccination. With the introduction of the pentavalent vaccine (P = MenABCWY), new schedule options are being considered. The aim of the current analysis is to evaluate the public health impact and cost-effectiveness of different meningococcal vaccination schedules vs. CVS.

**Methods:**

An incidence-based static population model focusing on a cohort of AYA (11 to 25 years of age) was used to compare multiple vaccination schedules at their steady state. Meningococcal vaccine type, effectiveness, vaccination coverage rate, age at administration (i.e., target population) and waning were considered for each modelled schedule to estimate age-dependent level of protection. The analysis was conducted from a societal perspective, including productivity losses due to IMD and IMD-related sequelae (in patients and caregivers), income lost due to premature death, and direct medical costs. The primary focus was on comparisons of the alternative schedules against CVS.

**Results:**

Under the current adolescent vaccination scheme (Q-QB-B) initiated at 11-12 years of age, 50 IMD cases (10.9 CWY, 39.1 B) occur per year. When the 2nd dose of Q and the 1st dose of B at age 16 is replaced with the P (Q-P-B), 1.5 cases are avoided and the ICER is 4.5 million vs CVS. When the1st and the last dose of B are replaced by a P (Q-P-P), 1.6 cases are avoided with an ICER of 6.8 million vs. CVS. If the 1st dose of Q is removed at 11-12 years from Q-P-P (N-P-P), total cost is reduced but 13.6 additional cases would occur every year.

**Conclusion:**

None of the scenarios in which the 2nd dose of MenACWY at 16 years of age was replaced by MenABCWY were cost-effective vs CVS. While cost savings could be realized through omitting the first MenACWY dose at 11-12, this is estimated to result in an increase in IMD cases with associated mortality and morbidity.

**Disclosures:**

Thomas Shin, MA, MPH, Sanofi: Employee of Sanofi|Sanofi: employee and may hold stock or stock options Olivier Cristeau risteau, DUT, Clever-Access: Employee and Clever-Access received funding from Sanofi to conduct this study Emilie Clay, PhD, MS, Clever-Access: employee|Clever-Access: Clever-Access received funding from Sanofi to conduct this study|Clever-Access: Clever-Access received funding from Sanofi to conduct this study|Clever-Access: Employee and Clever-Access received funding from Sanofi to conduct this study Angie Upegui, PhD, Sanofi: employee and may hold stock or stock options Rachel Dawson, DO, MPH, Sanofi: employee and may hold stock or stock options David P. Greenberg, MD, FIDSA, FAAP, Sanofi: employee and may hold stock or stock options Maureen P. Neary, PhD, MS, Sanofi: employee and may hold stock or stock options

